# The Interaction between Adult Cardiac Fibroblasts and Embryonic Stem Cell-Derived Cardiomyocytes Leads to Proarrhythmic Changes in* In Vitro* Cocultures

**DOI:** 10.1155/2016/2936126

**Published:** 2016-01-05

**Authors:** Jan Trieschmann, Daniel Bettin, Moritz Haustein, Annette Köster, Marek Molcanyi, Marcel Halbach, Mira Hanna, Mariam Fouad, Konrad Brockmeier, Jürgen Hescheler, Kurt Pfannkuche, Tobias Hannes

**Affiliations:** ^1^Department of Paediatric Cardiology, Heart Centre Cologne, University Hospital of Cologne, Kerpener Strasse 62, 50937 Cologne, Germany; ^2^Institute for Neurophysiology, University of Cologne, Robert-Koch-Strasse 39, 50931 Cologne, Germany; ^3^Department of Paediatrics, University Hospital of Cologne, Kerpener Strasse 62, 50937 Cologne, Germany; ^4^Department of Internal Medicine III, Heart Centre Cologne, University of Cologne, Kerpener Strasse 62, 50937 Cologne, Germany; ^5^Physiology Department, Faculty of Medicine (Kasr El-Aini), Cairo University, El-Maniel, Cairo 11451, Egypt

## Abstract

Transplantation of stem cell-derived cardiomyocytes is one of the most promising therapeutic approaches after myocardial infarction, as loss of cardiomyocytes is virtually irreversible by endogenous repair mechanisms. In myocardial scars, transplanted cardiomyocytes will be in immediate contact with cardiac fibroblasts. While it is well documented how the electrophysiology of neonatal cardiomyocytes is modulated by cardiac fibroblasts of the same developmental stage, it is unknown how adult cardiac fibroblasts (aCFs) affect the function of embryonic stem cell-derived cardiomyocytes (ESC-CMs). To investigate the effects of aCFs on ESC-CM electrophysiology, we performed extra- and intracellular recordings of murine aCF-ESC-CM cocultures. We observed that spontaneous beating behaviour was highly irregular in aCF-ESC-CM cocultures compared to cocultures with mesenchymal stem cells (coefficient of variation of the interspike interval: 40.5 ± 15.2% versus 9.3 ± 2.0%, *p* = 0.008) and that action potential amplitude and maximal upstroke velocity (*V*_max_) were reduced (amplitude: 52.3 ± 1.7 mV versus 65.1 ± 1.5 mV, *V*_max_: 7.0 ± 1.0 V/s versus 36.5 ± 5.3 V/s), while action potential duration (APD) was prolonged (APD50: 25.6 ± 1.0 ms versus 16.8 ± 1.9 ms, *p* < 0.001; APD90: 52.2 ± 1.5 ms versus 43.3 ± 3.3 ms, *p* < 0.01) compared to controls. Similar changes could be induced by aCF-conditioned medium. We conclude that the presence of aCFs changes automaticity and induces potentially proarrhythmic changes of ESC-CM electrophysiology.

## 1. Introduction

Cell transplantation is one of the most promising therapeutic approaches after myocardial infarction, as loss of cardiomyocytes is virtually irreversible by endogenous repair mechanisms.

Pluripotent stem cell-derived cardiomyocytes (PSC-CMs) seem particularly suited for this purpose, as they share many properties with native cardiomyocytes. One hallmark feature of PSC-CMs is their ability to electrically couple with host cardiomyocytes [1, 2]. Electrical coupling between host and transplanted cardiomyocytes drives maturation of the transplanted cardiomyocytes towards the electrical phenotype of the host cardiomyocytes [3–5] and it reduces postinfarction arrhythmias [1, 6].

Electrical coupling in the heart does not exclusively occur between cardiomyocytes. There is evidence for electrical coupling between cardiomyocytes and cardiac fibroblasts [7]. This heterocellular coupling to fibroblasts via gap junctions has been conclusively demonstrated in the sinoatrial node [8, 9]. Cardiomyocyte-fibroblast coupling might be involved in pathological conditions; for example, it could contribute to impulse propagation across scar borders [10–12].

Of note, there is evidence that coupling with cardiac fibroblasts might be able to modulate the electrophysiological properties of cardiomyocytes [13]. In a computational model of cardiomyocyte-fibroblast coupling, action potential duration is prolonged and resting membrane potential of cardiomyocytes is depolarised depending on the number of coupled fibroblasts [14]. Experimentally, a reduction of upstroke velocity in strands of neonatal cardiomyocytes was associated with higher fibroblast density [15]. These findings indicate that a high number of fibroblasts coupled to cardiomyocytes could induce changes that are associated with increased arrhythmogenicity.

In addition to coupling-induced effects, cardiac fibroblast can modulate cardiomyocyte electrophysiology [16] and Ca^2+^-handling [17] by paracrine signalling. Soluble factors secreted by neonatal cardiac fibroblasts prolonged action potential duration, depolarised the resting membrane potential, and slowed down upstroke velocity and conduction velocity of neonatal rat cardiomyocytes [16]. As these changes are potentially proarrhythmic, cardiac fibroblasts might be able to increase the likelihood of arrhythmias by paracrine signalling.

If transplanted into a scar region, PSC-CMs might be exposed to a high degree of electrical coupling to cardiac fibroblasts as well as paracrine factors secreted by adult cardiac fibroblasts. Given their relatively immature electrophysiological properties, the impact of fibroblast coupling on the electrical function of PSC-CMs is hardly predictable. In a previous study it was shown that neonatal cardiac fibroblasts are able to decrease the beating frequency and to increase the beating rate variability of PSC-CMs by coupling-dependent and coupling-independent mechanisms [18]. Addition of PSC-CMs to transforming growth factor-*β* treated monolayers of neonatal rat ventricular cells resulted in an increased conduction velocity and a reduced incidence of reentrant waves [19] was also shown to be coupling-dependent. Currently—to our knowledge—the effects of the interaction between PSC-CMs and adult cardiac fibroblasts have not yet been reported.

In addition to cardiac fibroblasts, mesenchymal stem cells (MSCs) have been shown to modulate the electrophysiological function of neonatal cardiomyocytes by coupling-dependent and coupling-independent mechanisms. Proarrhythmic effects of MSCs on neonatal ventricular rat myocytes have been amply documented [20, 21]; however, little is known on the effects of MSCs on the electrophysiological function of PSC-CMs. This should, however, be further investigated, since MSCs have been observed to support the engraftment of PSC-CMs in an* in vitro* cell transplantation model [22].

To investigate the effect of adult cardiac fibroblasts on the electrophysiological properties of embryonic stem cell-derived cardiomyocytes (ESC-CMs), we cocultured purified murine ESC-CMs with cultured murine primary adult cardiac fibroblasts (aCFs). We studied the regularity of the spontaneous electrical activity using microelectrode arrays (MEAs). To further understand the electrophysiological effects of MSCs on PSC-CMs, we compared aCF-ESC-CM cocultures with ESC-CMs cocultured with MSCs. In addition, we used sharp electrodes to record the transmembrane potential in aCF-ESC-CM cocultures and compared action potential parameters to ESC-CMs alone, ESC-CMs cultured in aCF-conditioned medium, and MSC-ESC-CM cocultures.

## 2. Materials and Methods

### 2.1. Isolation and Culture of Adult Cardiac Fibroblasts (aCFs)

Adult cardiac fibroblasts were obtained from male SV129/ola mice in two independent isolation procedures and were expanded* in vitro* afterwards. Hearts were excised after cervical dislocation and thoracotomy. Ventricular tissue was dissected, washed, minced, and subjected to seven times repeated digestions at 37°C for 20 minutes in a solution containing a mixture of 1 mg/mL of collagenase A and 0.5 mg/mL hyaluronidase upon an initial digestion step in a proteinase bacterial solution (4 U/mL) for 15 minutes. After each cycle of digestion, tissue was mechanically dissociated using a wide mouth pipet, the supernatant containing dissociated cells was collected, and cells were resuspended in Iscove's Modified Dulbecco's Medium (IMDM). Cells from all digestions were pooled and resuspended in IMDM supplemented with 20% foetal calf serum (FCS), penicillin (100 units/mL), streptomycin (100 *μ*g/mL), nonessential amino acids (1%), and 2-mercaptoethanol (0.1 mM). Cells were plated and incubated for 2 h to allow for the preferential attachment of fibroblasts. The supernatant was then replaced with fresh culture medium. For passage, aCFs were washed with D-PBS, dissociated with trypsin-EDTA (0.05%), and plated on culture dishes every week. aCFs were used for experiments between passages 3 and 10. If not stated otherwise, media and supplementals were purchased from Life Technologies (Life Technologies GmbH, Darmstadt, Germany).

### 2.2. Culture and Differentiation of Embryonic Stem Cells (ESCs)

Embryonic stem cells (ESCs) modified with a construct carrying a puromycin-acetyltransferase and eGFP under the control of the *α*-myosin heavy chain promoter (cell line D3/*α*PIG44 [23]) were maintained on inactivated murine embryonic fibroblasts (MEFs) in IMDM supplemented with FCS (15%), penicillin (100 U/mL), streptomycin (100 *μ*g/mL), nonessential amino acids (1%), 2-mercaptoethanol (0.1 mM), and Leukaemia Inhibitory Factor (1,000 U/mL). Every other day, ESCs were washed with D-PBS, trypsinised, centrifuged, and diluted to 5 × 10^5^ cells/dish.

For differentiation, 10^6^ cells were diluted to 14 mL IMDM supplemented with FCS (20%), penicillin (100 U/mL), streptomycin (100 *μ*g/mL), nonessential amino acids (1%), and 2-mercaptoethanol (0.1 mM). Formation of embryoid bodies (EBs) was induced by two days of shaking. Subsequently, EBs were diluted to 1,000/14 mL.

The presence of *α*-MHC-positive cells was checked for after 9 days and puromycin (7.5 *μ*g/mL) was added for purification. Puromycin was added a second time after 12 days of differentiation. ESC-CMs were used for all experiments at day 14 of differentiation.

### 2.3. Culture of Mesenchymal Stem Cells (MSCs)

MSCs were isolated from femurs of adult mice (strain: C57BL/6N). Cells were cultured in low glucose (1 g/L) Dulbecco's Modified Eagle Medium supplemented with FCS (15%), penicillin (100 U/mL), streptomycin (100 *μ*g/mL), nonessential amino acids (1%), and 10 ng/mL basic fibroblast growth factor (bFGF; PeproTech, Hamburg, Germany). After reaching 80–90% confluence, the cells were washed with D-PBS, detached with trypsin-EDTA (0.05%), and transferred onto new dishes or used for coculture experiments.

### 2.4. Multielectrode Array (MEA) Recordings

Since especially aCFs are highly mechanosensitive [8], we used multielectrode array (MEA) dishes to investigate the effects of MSCs and aCFs on the spontaneous activity of ESC-CMs. In contrast to the microelectrode measurement we used for the recording of transmembrane potentials, this method completely avoids mechanical manipulation before and during the recording. For MEA recordings, ESC-CMs were plated on gelatine-coated (0.1%) MEAs (Multichannel Systems, Reutlingen, Germany) at a density of 50,000 cells/dish together with aCFs or MSCs at a density of 10,000 cells/dish. We used MEAs with electrode diameters of 30 *μ*m and interelectrode distances of 200 *μ*m. Measurements with a duration of 10 minutes at a sampling frequency of 5 kHz were performed after two days of coculture using a MEA 1060 amplifier. Temperature was set to 37°C. Data were analysed offline using custom-written routines in MATLAB (The Mathworks, Natick, MA, USA). Only one electrode channel per coculture was analysed.

### 2.5. Microelectrode Measurements

For microelectrode measurements, we used spheroid aggregates of ESC-CMs and aCFs or MSCs and purified ESC-CM beating clusters as controls. For the formation of spheroid aggregates, ESC-CM beating clusters were trypsinised briefly to single cells (trypsin 0.25% approximately 10–15 minutes). The cell suspension was mixed with dissociated aCFs or MSCs (ESC-CMs 50,000/mL, aCFs or MSCs 10,000/mL if not indicated otherwise). Hanging drops (20 *μ*L) were formed on the top lid of a bacteriological dish containing 5 mL D-PBS. After two days, hanging drops were washed off. To determine the ratio between ESC-CMs and aCFs in the aggregates after culture, aCFs were stained with Vybrant DiI cell-labelling solution (Life Technologies) prior to aggregate formation (six independent cultures for each tested seeding ratio containing ~50 spheroid aggregates per culture). Cells were dissociated with trypsin 0.25% for 10 min (37°C) and stained with Hoechst for 5 min. GFP^+^/Hoechst^+^ cells were counted as ESC-CMs, while GFP^−^/Hoechst^+^ cells were counted as aCFs. For images of the aggregate structure, aCFs were stained with Vybrant DiI for 10 minutes before aggregate formation and images were acquired using an Axiovert 200 M equipped with the Zeiss ApoTome using Axiovision Release 4.4 (both Carl Zeiss, Jena, Germany).

APs were recorded with microelectrodes pulled from borosilicate capillaries (WPI, Sarasota, USA) filled with 3 M KCl solution at resistances between 30 and 60 MΩ. The signal was amplified by a SEC-10LX (npi electronics, Tamm, Germany) and digitised with a HEKA EPC-9 controlled by the Pulse software (HEKA Systems, Lambrecht/Pfalz, Germany). Action potentials were analysed using the MiniAnalysis software (Synaptosoft, Fort Lee, NJ, USA). One ESC-CM per aggregate or ESC-CM cluster was measured and analysed.

### 2.6. aCF-Conditioned Medium Experiments

To establish conditions similar to the previous microelectrode experiments, purified ESC-CM beating clusters at day 14 of differentiation were collected and cultured in aCF-conditioned medium or IMDM supplemented with 20% FCS for two more days. For medium conditioning, aCFs (100,000) were plated and cultured in 10 mL IMDM supplemented with FCS (20%) until they reached ~90% confluency after 7 days.

### 2.7. Immunohistochemistry

For histology, aCFs alone or cocultures with 50,000 ESC-CMs and 10,000 aCFs or MSCs were plated onto gelatine-coated (0.1%) coverslips. After 2 days, the preparations were fixed with methanol (−20°C, 5 min). Noteworthy, methanol effectively bleaches eGFP. Cells were rehydrated with D-PBS followed by blocking with Roti-Block (Carl Roth GmbH & Co. KG, Karlsruhe, Germany) for 1 hour. Incubations with anti-sarcomeric-*α*-actinin (Sigma, clone EA53, 1 : 800), anticonnexin 43 (Sigma, rabbit, polyclonal, C6219, 1 : 400), antivimentin (Sigma, mouse, clone VIM-13.2, IgM, V5255; 1 : 200), and anti-smooth muscle actin (Sigma, mouse, clone 1A4, IgG2a, A2547, 1 : 500) were done overnight at 4°C in 1% BSA in PBS. Secondary antibodies (anti-rabbit-AlexaFluor 488, anti-mouse-IgM-AlexaFluor 555, anti-mouse-IgG1-AlexaFluor 647, and anti-mouse-IgG2a-AlexaFluor 647 (all Life Technologies, 1 : 1000)) were applied for 60 min at room temperature. Nuclei were stained using Hoechst 33342 (Sigma-Aldrich Chemie GmbH, Steinheim, Germany). After washing, samples were embedded in ProLong Gold Antifade Reagent (Life Technologies). Images were acquired using an Axiovert 200 M equipped with the Zeiss ApoTome using Axiovision Release 4.4 (both Carl Zeiss, Jena, Germany).

### 2.8. Statistical Analysis

Data were analysed using IBM SPSS statistics 22 (IBM, Armonk, NY, USA). If not stated otherwise, values are presented as mean ± standard error of the mean (SEM). Statistical analysis was performed using unpaired *t*-test or where appropriate one-way ANOVA followed by Tukey-HSD or Dunnett T3* post hoc* test. Statistical significance was assumed at *p* < 0.05.

## 3. Results

### 3.1. aCFs Acquired and Maintained a Myofibroblast Phenotype and Expressed Homo- and Heterocellular Cx43 in Culture

**Figure 1:**
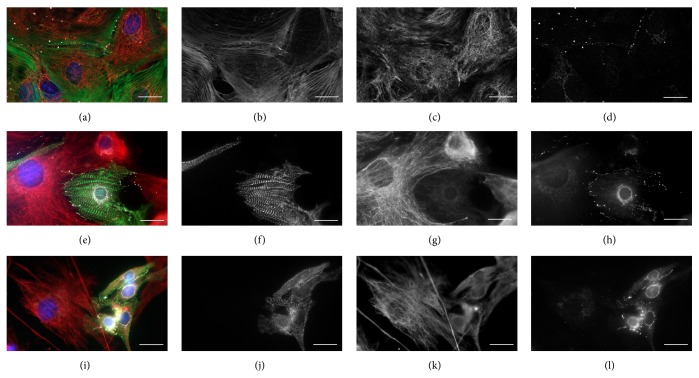
Cultured aCFs showed a myofibroblast phenotype and distribution of Cx43 in cocultures of ESC-CMs with aCFs and MSCs. Cultured aCFs (a–d), ESC-CMs, and aCFs after coculture (e–h) and ESC-CMs and MSCs after coculture (i–l). Merged picture of the different channels (a, e, i) as well as *α*-smooth muscle actinin (b), sarcomeric *α*-actinin (f, j), vimentin (c, g, k), and Cx43 (d, h, l). Scale bars = 20 *μ*m.

aCFs were cultured on gelatine-coated plastic dishes. Between passages 2 and 10, aCFs monocultures (Figure 1(a)) expressed *α*-SMA (Figure 1(b)) and vimentin (Figure 1(c)) indicating that aCFs transformed into a myofibroblast-like phenotype. We observed abundant Cx43-expression in aCF monocultures (Figure 1(d)) suggesting the presence of gap junctions between aCFs.

In cocultures with ESC-CMs and aCFs (Figure 1(e)), we found Cx43 at the borders between the *α*-actinin positive ESC-CMs (Figure 1(f)) and vimentin-positive aCFs (Figure 1(g)) suggesting the formation of heterocellular gap junctions (Figure 1(h)). Similarly, we observed Cx43 between ESC-CMs and MSCs (Figures 1(i)–1(l)).

### 3.2. Coculture with aCFs but Not MSCs Induced Irregular Beating of ESC-CMs

**Figure 2:**
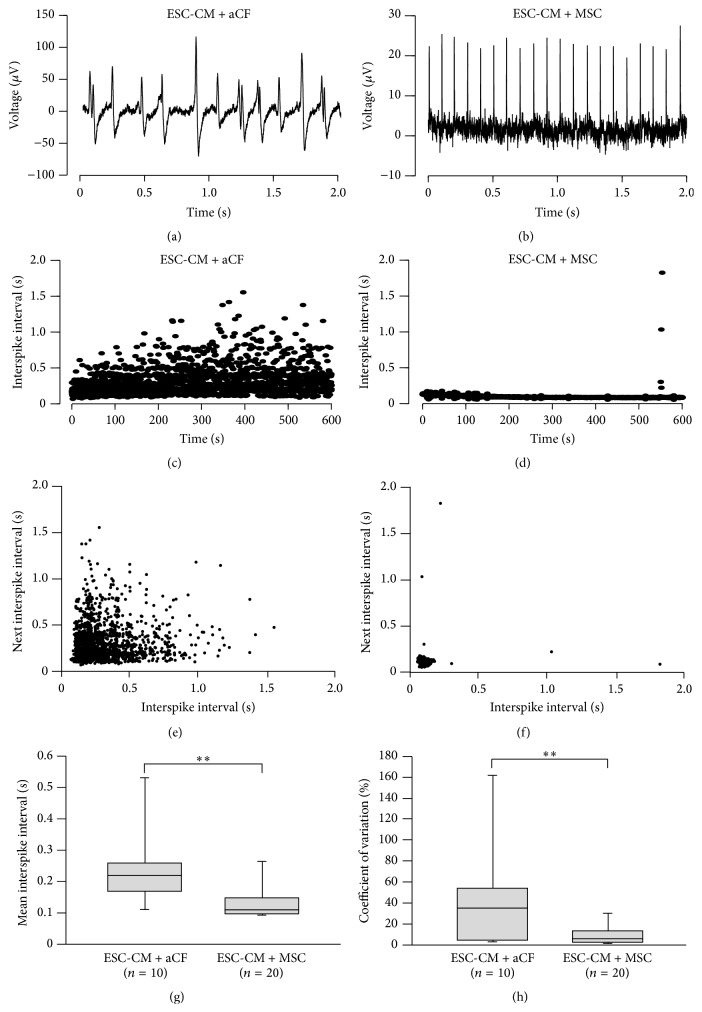
Effects of aCFs and MSCs on beating regularity of ESC-CMs during MEA measurements. Representative traces of aCF-ESC-CM (a) and MSC-ESC-CM (b) cocultures. ISIs of representative recordings of aCF-ESC-CM (c) and MSC-ESC-CM (d) cocultures. (e, f) Poincaré plots of representative measurements. No propagation block patterns could be identified as a potential cause of the increased beat-to-beat variability. Mean ISI was prolonged (g) and coefficient of variation was higher (h) in aCF-ESC-CM cocultures compared to MSC-ESC-CM cocultures. Data are expressed as mean ± SEM; ^*∗∗*^ *p* < 0.01.

MEA measurements were performed after 2 days of coculture with MSCs (eight independent experiments) or aCFs (three independent experiments). Cocultures with aCFs showed significantly longer interspike intervals (ISIs) compared to cocultures with MSCs (236.1 ± 37 ms, *n* = 10 versus 132.4 ± 12.2 ms, *n* = 20, *p* = 0.001) (Figure 2(e)).

We observed that cocultures with aCFs beat highly irregularly (Figures 2(a) and 2(c)), while MSC cocultures showed regular beating (Figures 2(b) and 2(d)). To express beating regularity quantitatively, we calculated the coefficient of variation of ISIs for each individual measurement. Corresponding to the high degree of irregularity, aCF cocultures showed a high coefficient of variation compared to cocultures with MSCs (40.5 ± 15.2%, *n* = 10 versus 9.3 ± 2.0%, *n* = 20, *p* = 0.008) (Figure 2(f)).

As a higher frequency of propagation block could be a potential mechanism contributing to the increased beat-to-beat variability, we used Poincaré plots (Figures 2(e) and 2(f)) to identify potential propagation block [24]. None of the measurements, however, showed propagation block patterns.

### 3.3. Coculture with aCFs Depolarised Resting Membrane Potential and Prolonged Action Potential Duration of ESC-CMs

**Figure 3:**
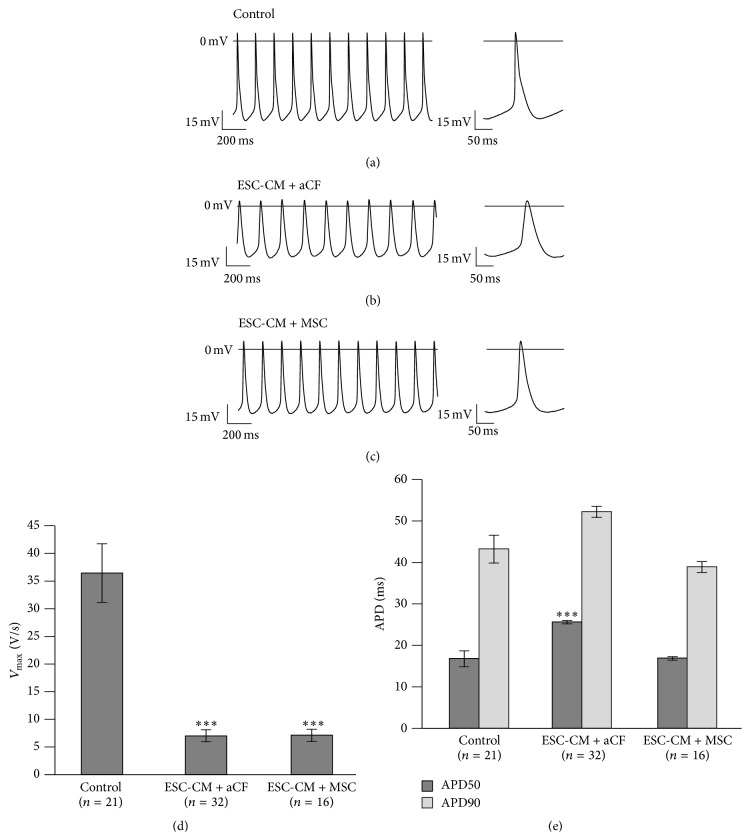
Comparison of action potential parameters of aCF-ESC-CM and MSC-ESC-CM cocultures as well as control ESC-CMs. Representative AP waveforms of beating clusters of ESC-CMs (a) as well as cocultures of ESC-CMs with aCFs (b) or with MSCs (c). Maximal upstroke velocity (*V*_max_) was reduced in cocultures with aCFs and MSCs (d) while action potential duration was only prolonged in cocultures of ESC-CMs with aCFs (e) compared to controls. Data are expressed as mean ± SEM; ^*∗∗∗*^ *p* < 0.001.

Compared to control ESC-CMs (five independent cultures) (Figure 3(a)), ESC-CMs in coculture with aCFs (five independent cultures) (Figure 3(b)) and with MSCs (six independent cultures) (Figure 3(c)) showed a different AP morphology. AP frequency was lower in aCF-ESC-CMs compared to control ESC-CMs (aCF cocultures, *n* = 32: 4.0 ± 0.3 Hz versus controls, *n* = 21: 5.4 ± 0.4 Hz, *p* = 0.015). The maximal upstroke velocity (*V*_max_) (Figure 3(d)) and amplitude were significantly reduced (*V*_max_: 7.0 ± 1.0 V/s versus 36.5 ± 5.3 V/s, *p* < 0.001; amplitude: 52.3 ± 1.7 mV versus 65.1 ± 1.5 mV, *p* < 0.001). ESC-CMs cocultured with aCFs showed a significantly more depolarised maximal diastolic potential (MDP) (−47.1 ± 1.5 mV versus −57.3 ± 1.7 mV, *p* < 0.001). APD50 was significantly prolonged, while the prolongation of APD90 did not reach significance (APD50: 25.6 ± 1.0 ms versus 16.8 ± 1.9 ms, *p* < 0.001; APD90: 52.2 ± 1.5 ms versus 43.3 ± 3.3 ms, *p* = 0.055) (Figure 3(e)).

To compare the effects with other cells able to form gap junctions with cardiomyocytes, we prepared hanging drop cocultures of ESC-CMs and MSCs. Amplitude and *V*_max_ (Figure 3(d)) were reduced in MSC cocultures (*n* = 16) compared to controls (*n* = 21) (amplitude: 49.4 ± 1.8 mV versus 65.1 ± 1.5 mV, *p* < 0.001; *V*_max_: 7.1 ± 1.1 V/s versus 36.5 ± 5.3 V/s, *p* < 0.001). Similar to ESC-CMs cocultured with aCFs, ESC-CMs cocultured with MSCs showed a significantly more depolarised MDP (−49.9 ± 1.4 mV versus −57.3 ± 1.7 mV, *p* = 0.016). In contrast to aCF cocultures, AP frequency was not decreased in MSC cocultures (6.7 ± 0.3 Hz versus controls: 5.4 ± 0.4 Hz, *p* = 0.076). APD50 and APD90 were not significantly changed compared to controls (APD50: 16.8 ± 1.9 versus 16.9 ± 0.4, *p* = 0.999; APD90: 38.9 ± 1.3 ms versus 43.3 ± 3.3 ms, *p* = 0.537).

Comparing MSC (*n* = 16) and aCF (*n* = 32) cocultures, APD50 and APD90 were significantly longer in aCF cocultures (APD50: 25.6 ± 1.0 ms versus 16.9 ± 0.4 ms, *p* < 0.001; APD90: 52.2 ± 1.5 ms versus 38.9 ± 1.3 ms, *p* < 0.001) (Figure 3(e)). Frequency was significantly lower in aCF cocultures (4.0 ± 0.3 Hz versus 6.7 ± 0.3 Hz, *p* < 0.001). Amplitude, *V*_max_, and MDP were not significantly different.

### 3.4. Electrophysiological Effects of aCF Coculture Were Dependent on aCF Density

**Figure 4:**
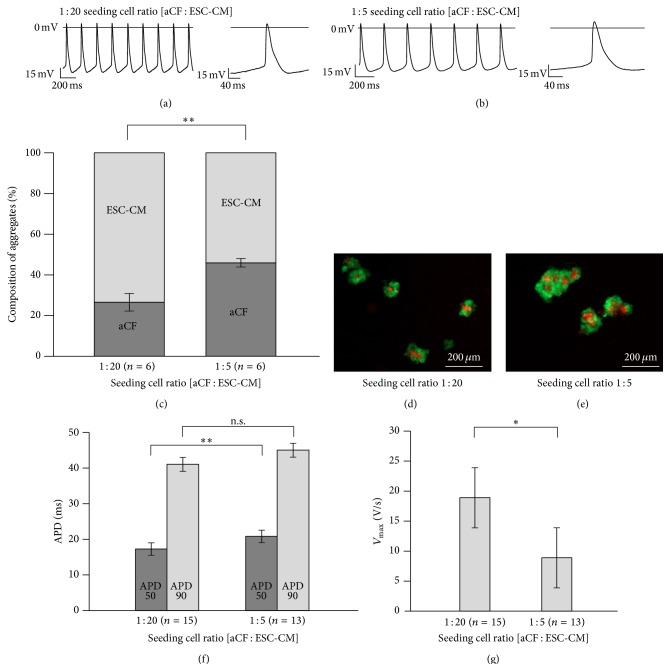
Impact of different seeding aCF densities on action potential recordings of cocultures with ESC-CMs. Representative AP waveforms of cocultures with a lower (1 : 20) (a) and higher (1 : 5) (b) seeding number of aCFs. Percentage of ESC-CMs and aCFs in hanging drops cocultured for two days (c). Pictures of cocultures with aCF seeding ratios 1 : 20 (d) and 1 : 5 (e) after two days in culture. Higher number of aCFs in cocultures resulted in a decreased maximal upstroke velocity (*V*_max_) (f) as well as a prolonged APD50 (g). Data are expressed as mean ± SEM; ^*∗*^ *p* < 0.05; ^*∗∗*^ *p* < 0.01.

To test whether the observed electrophysiological changes are dependent on the aCF density, we prepared hanging drops (five independent cultures) with a low (*n* = 15) and a high (*n* = 13) density of aCFs (1 : 20 seeding cell ratio: 50 aCFs/1,000 ESC-CMs; 1 : 5 seeding cell ratio: 200 aCFs/1,000 ESC-CMs) (Figures 4(e) and 4(d)). After two days, beating clusters were dissociated and cells were counted. The low density beating clusters contained 27 ± 4% aCFs and the high density beating clusters contained 46 ± 2% cardiomyocytes (*n* = 6 independent cocultures, *p* = 0.003) (Figure 4(c)). This corresponded to an aCF/ESC-CM ratio of 0.39 ± 0.09 versus 0.86 ± 0.06 (*p* = 0.002) after two days in culture.

A higher aCF density led to a mild prolongation of APD50 (1 : 20 seeding cell ratio: 17.3 ± 0.6 ms versus 1 : 5 seeding cell ratio: 20.8 ± 1.0 ms, *p* = 0.006) (Figure 4(g)) and a reduction of *V*_max_ (1 : 20 seeding cell ratio: 18.9 ± 3.3 V/s versus 1 : 5 seeding cell ratio: 8.9 ± 1.8 V/s, *p* = 0.018) (Figure 4(f)). MDP was mildly more depolarised in cocultures with a higher aCF density (1 : 20 seeding cell ratio: −50.4 ± 1.7 mV versus 1 : 5 seeding cell ratio: −54 ± 2.1 mV); this effect was however not significant (*p* = 0.197). In addition, frequency, amplitude, and APD90 were not significantly changed.

### 3.5. aCF-Conditioned Medium Prolonged APD50 and Reduced AP Frequency

**Figure 5:**
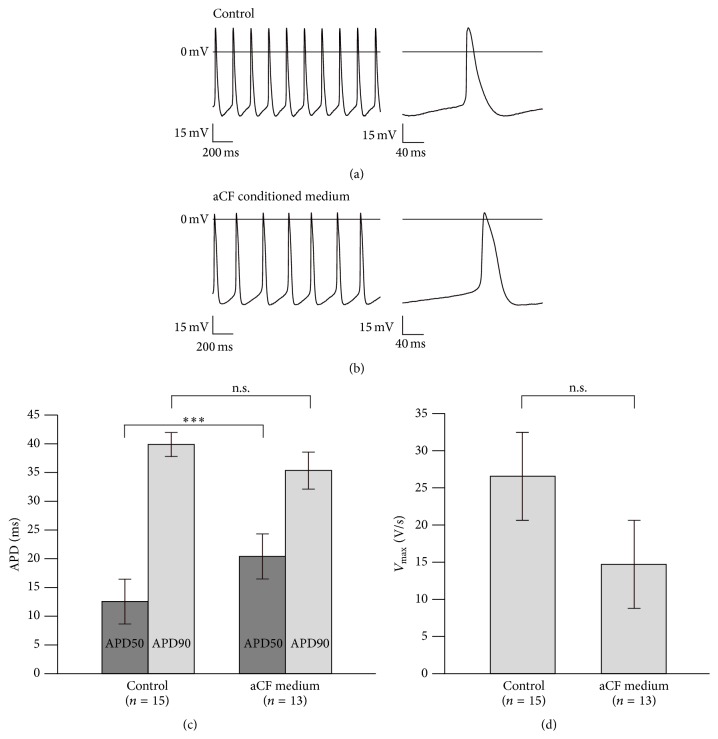
Effects of aCF-conditioned medium on action potential recordings of ESC-CMs. Representative AP waveforms of ESC-CM beating clusters cultured for 48 h in control medium (a) and aCF-conditioned medium (b). APD50 was prolonged (c). Maximal upstroke velocity (*V*_max_) was not significantly reduced (d). Data are expressed as mean ± SEM; ^*∗∗∗*^ *p* < 0.001.

We tried to estimate the effect of paracrine mediators on ESC-CM electrophysiology by comparing control ESC-CMs (Figure 5(a)) to ESC-CMs incubated with aCF-conditioned medium for 48 h (three independent experiments) (Figure 5(b)). After 48 h of incubation, APD50 was increased (aCF-conditioned medium, *n* = 13: 20.4 ± 1.6 ms versus controls, *n* = 15: 12.5 ± 1.0 ms, *p* < 0.001) (Figure 5(c)) and AP frequency was reduced (aCF-conditioned medium: 3.5 ± 0.3 Hz versus controls: 4.8 ± 0.4 Hz, *p* = 0.016) compared to untreated controls. In addition, amplitude was increased (aCF-conditioned medium: 64.2 ± 2.6 mV versus controls: 56.7 ± 2.2 mV, *p* = 0.037). *V*_max_ was not significantly reduced (aCF-conditioned medium: 14.7 ± 2.8 V/s versus controls: 26.6 ± 5.6 V/s, *p* = 0.081) (Figure 5(d)). MDP was not significantly changed (aCF-conditioned medium: −50.1 ± 3 mV versus controls: −54.1 ± 2.2 mV, *p* = 0.358).

## 4. Discussion

In the present study, we were able to show that (1) aCFs as well as MSCs are able to form heterocellular gap junctions with ESC-CMs, (2) coculture with aCFs induced irregular beating patterns in ESC-CMs, while coculture with MSCs did not, (3) action potential amplitude and *V*_max_ were reduced in ESC-CMs cocultured with aCFs and MSCs, and (4) APD was increased in ESC-CMs cocultured with aCFs, but not with MSCs. We could further (5) demonstrate that electrophysiological changes were more prominent at higher aCF densities. (6) Similar to cocultures with aCFs, action potential frequency was decreased and APD was prolonged in ESC-CMs incubated with aCF-conditioned medium, while *V*_max_ was however not significantly affected.

Spontaneous beating is a common feature of pluripotent stem cell-derived cardiomyocytes. Multiple underlying mechanisms have been well studied [25–32]. When cocultured with aCFs, spontaneous beating frequency of ESC-CMs decreased in comparison to cocultures with MSCs. This can be explained by the depolarisation of the resting membrane potential in aCF-ESC-CM cocultures which we observed in our sharp electrode recordings. Similar effects have been observed with primary and cultured cardiomyocytes. HL-1 cardiomyocytes or ESC-CMs cocultured with neonatal rat ventricular fibroblasts showed a decrease in spontaneous beating frequency which was dependent on Cx43-mediated electrical coupling [18]. The role of electrical coupling is further supported by the observation that resting membrane potential is dependent on fibroblast density in patterned cultures of neonatal rat cardiomyocytes [15]. In our study, however, aCF-conditioned medium was able to reduce AP frequency by approximately the same amount. It seems, therefore, that electrical coupling and subsequent depolarisation of the resting membrane potential as well as paracrine factors are able to reduce the AP frequency of ESC-CMs cocultured with fibroblasts.

Similar to ESC-CMs cocultured with aCFs, the resting membrane potential of ESC-CMs was depolarised when cocultured with MSCs. This again is most likely due to electrical coupling since MSCs are able to form heterocellular gap junctions with cardiomyocytes via Cx43 [21]. Instead of the expected reduction of beating frequency due to a more depolarised resting membrane potential, ESC-CMs cocultured with MSCs beat at a rate comparable to controls. By assuming that electrical coupling contributes similarly in aCF-ESC-CM and MSC-ESC-CM cocultures, the significant higher automaticity in MSC-ESC-CM cocultures might be mediated by differences in the secretome between aCFs and MSCs.

While suppression of automaticity is desirable for any potential effect of cell transplantation (with the exception of biological pacemakers), the reduced excitability could favour arrhythmogenicity after cell transplantation by slow conduction [33]. We therefore hypothesise that the interaction of PSC-CMs and aCFs might be able to contribute to posttransplantation arrhythmia.

Beyond a potential role of the interaction between stem cell-derived cardiomyocytes and aCFs in cell transplantation, our results point to an important role of aCFs for the regulation of beat-to-beat variability. When cocultured with MSCs, we observed only a very small beat-to-beat variability. This is in line with the previously reported low beat-to-beat variability for this cell line [34]. However, beat-to-beat variability is clearly a physiological feature of cardiac tissue and is well preserved in monolayer cultures [35] and in cardiomyocytes derived from various other stem cell lines [34, 36]. In our study, the coculture with aCFs increases beat-to-beat variability, but the induction of other potentially deleterious changes in ESC-CM electrophysiology might be the result of a potential developmental “gap” between adult aCFs and immature ESC-CMs.

In addition to automaticity and excitability, also repolarisation of ESC-CMs was affected when cocultured with aCFs. In our study, only coculture with aCFs, but not with MSCs, prolonged APD50 significantly. We could further show that APD50 was more prolonged in cocultures with higher aCF density. Similarly, aCF-conditioned medium could induce a prolongation of APD50 in the absence of aCFs. This is in line with previous observations showing that paracrine factors alone are able to prolong APD of rat neonatal cardiomyocytes [16]. It is trivial to mention that changes in repolarisation can be proarrhythmic if they result in increased dispersion of repolarisation. Therefore, it can be speculated that aCF-mediated alterations of repolarisation might also contribute to potential proarrhythmic effects of cell transplantation.

We chose the seeding ratios similar to a previous study investigating the aggregation behaviour of ESC-CMs in the presence and absence of murine embryonic fibroblasts [37]. Here, ESC-CMs were able to reaggregate only in the presence of embryonic fibroblasts. In the present study, aCFs as well as MSCs were also able to facilitate reaggregation with the same seeding ratio and, in the case of cardiac fibroblasts, with a lower ratio.

It is, however, important to mention that the ratios between aCFs and ESC-CMs drastically changed within the short time of coculture. In the case of a 1 : 5 seeding ratio, we observed a ratio of approximately 1 : 1 after 48 hours. While proliferation doubling times of 28.9 hours for young adult rat cardiac fibroblasts [38] and 36 hours for human cardiac fibroblasts [39] have been reported, it is well known that ESC-CMs proliferate only at low rates [40]. We assume that the difference between seeding ratio and cell ratio at the time of measurements is due to nonadherence of cells in the hanging drop culture system, differential proliferative activity, and cell death.

For the translation to* in vivo* conditions, it would be important to approximate ratio and distribution of aCFs and MSC which can be achieved after cell transplantation into infarction scars. To our knowledge, tangible data on these two parameters have not yet been reported. Considering that in the native myocardium fibroblasts predominate quantitatively, our results suggest that marked electrophysiological effects occur in ESC-CMs with even lower fibroblast numbers.

In the present study, ESC-CMs showed alterations of their electrophysiological behaviour when cocultured with aCFs and—to a minor extent-with MSCs. The myocyte—fibroblast interaction seems to be extremely complex [41] involving many different soluble factors such as transforming growth factor-*β*, tumour necrosis factor-*α*, vascular endothelial growth factor, fibroblast growth factor-2, angiotensin-II, endothelin-1, or members of the interleukin family. In additions, fibroblasts and cardiomyocytes communicate via transformation of the extracellular matrix and direct cell-cell interactions involving electrical coupling via gap junctions as well as mechanical coupling via adherens junctions.

The complexity of the fibroblast-cardiomyocyte interaction suggests that a network of factors rather than single factors contributes to the observed changes. It will therefore be extremely demanding to decipher and to control the mechanisms leading to proarrhythmic changes in aCF-ESC-CM cocultures. We rather would like to suggest that avoiding transplantation of dissociated PSC-CMs into fibroblast-rich scars might prevent posttransplantation arrhythmias. This advocates the use of engineered heart tissue [42–44] rather than the direct transplantation of cardiomyocytes.

### 4.1. Limitations

As our study was conducted* in vitro*, it bears the risk of experimental artefacts and false interpolation of results to* in vivo* conditions. We believe that this is balanced out by the high degree of control over experimental variables such as cell numbers on the one hand. On the other hand, to our knowledge, there is no experimental data on the electrophysiological behaviour of PSC-CMs into ischaemic myocardial scars. Therefore, we hope that our data might help in anticipating problems that arise once repopulation of ischemia-induced myocardial scars with PSC-CMs becomes successful.

For the MEA measurements, we could only compare aCF-ESC-CM cocultures and MSC-ESC-CM cocultures, but not ESC-CMs alone as controls, since ESC-CMs derived from the cell line we used for the present study are unable to quantitatively attach to noncoated or coated surfaces [37]. Thus, we cannot unequivocally exclude other factors compared to coupling-dependent and paracrine mechanisms.

We can only present immunohistochemical data on the distribution of connexin 43 in the aCF-ESC-CM and MSC-ESC-CM cocultures. We therefore can only speculate that electrical coupling might be involved in changing the electrophysiological properties of ESC-CMs by the presence of aCFs and MSCs.

## 5. Conclusions

We conclude from our results that the presence of aCFs changes automaticity and potentially induces proarrhythmic changes of ESC-CM electrophysiology such as reduced excitability and prolonged repolarisation. Although difficult to study* in vivo*, these effects need to be considered in translational approaches of cardiomyocyte transplantation.
